# Acceptability of Rapid Diagnostic Test-Based Management of Malaria among Caregivers of Under-Five Children in Rural Ghana

**DOI:** 10.1371/journal.pone.0045556

**Published:** 2012-09-18

**Authors:** Frank Baiden, Seth Owusu-Agyei, Eunice Okyere, Mathilda Tivura, George Adjei, Daniel Chandramohan, Jayne Webster

**Affiliations:** 1 Malaria Group, Kintampo Health Research Centre, Kintampo, Ghana; 2 Social Science Group, Kintampo Health Research Centre, Kintampo, Ghana; 3 Statistics Unit, Kintampo Health Research Centre, Kintampo, Ghana; 4 Department for Disease Control, London School of Hygiene and Tropical Medicine, London, United Kingdom; 5 Department for Disease Control, London School of Hygiene and Tropical Medicine, London, United Kingdom; Kenya Medical Research Institute – Wellcome Trust Research Programme, Kenya

## Abstract

**Introduction:**

WHO now recommends test-based management of malaria (TBMM) across all age-groups. This implies artemisinin-based combination treatment (ACT) should be restricted to rapid diagnostic test (RDT)-positive cases. This is a departure from what caregivers in rural communities have been used to for many years.

**Methods:**

We conducted a survey among caregivers living close to 32 health centres in six districts in rural Ghana and used logistic regression to explore factors likely to influence caregiver acceptability of RDT based case management and concern about the denial of ACT on account of negative RDT results. Focus group discussions were conducted to explain the quantitative findings and to elicit further factors.

**Results:**

A total of 3047 caregivers were interviewed. Nearly all (98%) reported a preference for TBMM over presumptive treatment. Caregivers who preferred TBMM were less likely to be concerned about the denial of ACT to their test-negative children (O.R. 0.57, 95%C.I. 0.33–0.98). Compared with caregivers who had never secured national health insurance cover, caregivers who had valid (adjusted O.R. 1.30, 95% CI 1.07–1.61) or expired (adjusted O.R. 1.38, 95% CI 1.12–1.73) insurance cover were more likely to be concerned about the denial of ACT to their RDT-negative children. Major factors that promote TBMM acceptability include the perception that a blood test at health centre level represents improvement in the quality of care, leads to improvement in treatment outcomes, and offers opportunity for better communication between health workers and caregivers. Acceptability is also enhanced by engaging caregivers in the procedures of the test. Apprehensions about negative health worker attitude could however undermine acceptance.

**Conclusion:**

Test (RDT)-based management of malaria in under-five children is likely to be acceptable to caregivers in rural Ghana. The quality of caregiver-health worker interaction needs to be improved if acceptability is to be sustained.

## Introduction

Presumptive management of malaria was practiced for many years in low-income countries because of the lack of laboratory facilities, the availability of cheap antimalarials and the need to avert deaths through early initiation of treatment [1], [2]. The approach however led to the over-diagnosis of malaria and the overuse of antimalarials, with attendant development of strains of the parasites that are resistant to previously-used antimalarials [3], [4], [5], [6]. In 2010, The World Health Organisation (WHO) issued revised malaria treatment guidelines that recommend test-based management of malaria in all transmission settings and across all age-groups. For practical reasons, rapid diagnostic tests (RDTs) will be the main tool for implementing test-based management of malaria [7]. The decision to revise the malaria treatment guidelines was not without considerable debate. Arguments in favour of the shift to test-based management of malaria include: malaria transmission has been declining in areas previously considered to be very high; due to emergence of resistance cheap antimalarials have been replaced with the relatively more expensive artemisinin-based combination therapy (ACT); smear microscopy is no longer the only means of confirming the diagnosis of malaria since accurate and reliable rapid diagnostic tests (RDTs) are available; a policy of test-based management of malaria will lead to improvement in the management of non-malaria febrile illnesses within the context of IMCI [8], [9].

Conversely, arguments opposing the shift to test-based management of malaria have included: there is insufficient evidence that malaria transmission is declining and will remain on the decline; the inadequacy of the health systems in malaria-endemic countries to ensure continuous availability of quality-assured RDTs; there is insufficient evidence on the safety of restricting ACT to test-positive cases; and that a policy of test-based management of malaria will not necessarily lead to improvement in the management of non-malarial febrile illnesses [10], [11], [12].

For the policy of test-based management of malaria to be applied in under-five children, it must be incorporated into the Integrated Management of Childhood Illnesses (IMCI) [8], [13]. A critical factor in the success of this strategy is how caregivers of under-five children perceive test-based management of malaria which represents a change in how their febrile children are diagnosed, and whether they consider it acceptable. In the era of presumptive treatment of malaria, case management guidelines within the Integrated Management of Childhood Illnesses (IMCI) required that all under-five children with fever in high-transmission settings should be prescribed an antimalarial [14]. As part of the strategy of home management for malaria, caregivers were encouraged to initiate presumptive treatment for malaria at home before sending sick children to the health facility [15], [16]. Presumptive treatment thus became an established practice in the management of malaria and the prescription of antimalarial became a part of the expectations of caregivers attending primary care facilities in endemic countries [17], [18], [19]. In the emerging era of test-based management of malaria however, fewer febrile children will be confirmed to have malaria and therefore prescribed antimalarials. Prescriptions for artemisinin-based combination treatments (ACT) could be reduced by as much as 50–70% [2], [20]. This means that test negative febrile children will be denied an ACT and there is little understanding of how this will influence the behaviour of caregivers. It is possible that this may lead to alternative ACT-seeking behaviour as caregivers denied ACTs seek to access them from other sources. Using an RDT to test for malaria requires that a blood sample is taken whenever a febrile child presents at a health facility. The practice of drawing blood to perform tests in primary care settings would be a marked departure from what caregivers' have experienced for many years.

Ghana has an estimated population of 24.2 million and an annual population growth rate of 2.4%. (http://www.ghana.gov.gh/census/phc2010.pdf). It has a gross domestic product per capita of US$1,325. Life expectancy at birth is estimated at 63.8 years. Infant mortality and under-five mortality rates are estimated at 50 and 111 deaths per 1000 live births respectively [21], [22]. The entire area of Ghana is considered to be hyper-endemic for malaria. Transmission is all-year round, but particularly high during the rainy seasons. According to facility-based data from the Ghana Health Service (GHS), malaria is the leading cause of morbidity in the country [23]. In Ghana the district is the operational unit of the health system. The highest referral facility at the district level is the district hospital. Health centres exist at sub-district level and are the lowest level of institutional care. They often lack laboratory facilities except where they are large and upgrading to the status of hospitals is planned [24]. Clinical care, including the management of malaria in most health centres in the country is therefore based on the presumptive approach.

In Ghana, the revised IMCI guidelines that incorporate RDT-based diagnosis and management of malaria are currently being introduced, replacing those including the presumptive approach. The new guidelines are for the clinical care of under-five children in health centres and other clinical care settings that similarly lack laboratory facilities. Although a number of studies have assessed the potential to incorporate RDT-based management of malaria into clinical care in Ghana [25], [26], no study has as yet assessed how caregivers will perceive the new approach.

A large, cluster randomised-controlled trial evaluating the effect of restricting ACT to RDT-positive cases in under-five children is currently ongoing in six districts of the Brong Ahafo Region of Ghana. The trial (registered at www.clinitrials.gov as NCT00832754) has a cohort of 3063 children attending 16 health centres that are implementing test (with RDT)-based management of malaria and 16 other health centres that are implementing the presumptive approach. We took advantage of this trial to embed a study to assess the acceptability of RDT-based management of malaria among caregivers of children enrolled in the trial. The study, which we report here, involved a combination of quantitative (survey using a structured questionnaire) and qualitative (focus group discussions-FGDs) approaches.

## Methods

### Ethics statement

Written informed consent was obtained from all caregivers before participation in the survey and FGDs. Permission to extract and publish anonymous quotations from caregiver comments in this paper was also sought. The protocol for the study was approved by the Ethics Review Committees of the Kintampo Health Research Centre, the Ghana Health Service and the London School of Hygiene and Tropical Medicine.

### Study setting

The study was undertaken in the Brong Ahafo Region of Ghana. It involved 6 out of the 22 districts in the region. These are the Kintampo North, Kintampo South, Nkoranza North, Nkoranza South, Tain and Techiman districts. All six districts are located within the forest-savannah transition zone of the country and were selected on the basis of the long-standing working relationship between the health authorities and communities within the districts, and the KHRC. Malaria transmission is high in all six districts with entomological inoculation rate (EIR) estimated at 269 infective bites per person per year [27]. Malaria is the leading cause of under-five out-patient attendance in all health facilities in the six districts [28].

### Survey (Quantitative component)

Prior to the commencement of the clinical trial, caregivers of children less than two years of age, living within 2 kilometres of 32 health centres in the six districts were interviewed in their homes by trained field supervisors, using a structured questionnaire. The health centres were selected using probability proportional to out-patient attendance in the year preceding the survey. The list of caregivers of potentially-eligible children was generated from the database of the KHRC Demographic Surveillance System (DSS) using simple random sampling. The target was to interview 100 caregivers per health centre, and to enrol their children into the clinical trial.

The questionnaire consisted of structured questions on the demographic characteristics, household assets, household characteristics (related to water supply, sanitation and type of fuel), ITN ownership and use, and acceptability of RDT-based management of malaria. The household assets and characteristics used in the questionnaire were similar to those used in the 2008 Ghana Demographic and Health Survey [29]. The question that was used to assess the acceptability of RDT-based management of malaria was: “There is now a rapid test that can tell if a child's fever is caused by malaria or other infection. Would you prefer that the doctor uses the test to decide whether to give malaria treatment or not, or to use what you tell him/her and what he/she finds on touching the child?” Two related follow-up questions were: (1) “Doing the test will mean that each time the child has fever some little (finger prick) blood will be taken to do the test. Would you still prefer RDT-based malaria treatment?” (2) “How worried would you be when you think or know that your child's fever is malaria but the doctor does not give malaria treatment?” The questionnaire was pre-tested among caregivers in facilities which were not part of the study. It was revised, finalised and administered just prior to commencement of the randomised-controlled trial. The survey was conducted between February 2010 and June 2010. This was over a time when RDT-based management of malaria had not been introduced into the health centres and there was little caregiver experience with its implementation.

### Survey sample size

A total of 3200 caregivers were targeted to be interviewed. This estimation was based upon statistical considerations that were made for the clinical trial. With this sample size however, it was possible to estimate the level of acceptability of RDT-based management of malaria based upon responses to the questions with a precision of 1.5%, at 95% confidence level, given a population of about 10,000 eligible caregivers and assuming a level of acceptability of RDT-based management of malaria of 50%.

The data was doubled-entered, validated and cleaned using FoxPro version 9.0 and exported into Stata version 11 for analysis. Using data on 11 household assets and 5 characteristics of the dwelling of caregivers, principal component analysis was used to generate wealth quintiles [30]. The quintiles classified each caregiver as being from households that were “least poor” “poor”, “very poor”, “most poor” or “poorest”. Descriptive analysis was performed using frequency functions. Univariate analysis was performed to identify significant predictors of acceptability of RDT-based management of malaria and ACT denial based upon an RDT-negative result. Statistically significant variables from the univariate analysis were adjusted in logistic regression models to determine independent predictors of the two outcomes of interest.

### Focus Group Discussions (Qualitative component)

Twelve to fifteen months (between June 2010 and August 2010) into the implementation of the trial, we conducted FGDs with caregivers of children enrolled in the trial to assess the acceptability of RDT-based management of malaria. Participants were caregivers of children in the intervention arm of the trial who had visited one of the study health centres in the preceding 6 weeks. As a part of the protocol of the clinical trial all children in the intervention arm had rapid tests for malaria and haemoglobin levels performed when they attended health facilities. In order to obtain as varied a perspective as possible, caregiver FGDs were formed according to the results of RDTs on their children (positive, negative and mix of both) and the locations of their residences (rural or semirural). Forty-six caregivers participated in the FGDs, with each FGD comprising 6 to 8 participants.

Factors that emerged from preliminary analysis of the survey data such as the apparently high caregiver acceptance of RDT-based management and the reaction towards ACT denial following a test-negative result were included in the FGD topic guide. Other related behavioural and contextual issues that emerged in the course of the clinical trial were also included in the guide. Exploring the influence of health insurance status on attitudes towards the denial of ACT (following an RDT negative result) was inadvertently excluded from the FGD guide and this is a limitation of the study. The guide was piloted among women living outside the study area and therefore not participants in the trial or FGDs. All the FGDs were conducted in convenient locations within the communities; away from the health facilities. They were conducted in the local dialect and moderated by experienced social scientists from the Kintampo Health Research Centre. Caregivers did not know the moderators prior to the day of interview and they were only introduced as workers of the research centre who had come to facilitate the discussions. Each item on the FGD guide was explored to the point when no new issues emerged.

All the FGDs were tape-recorded, transcribed and translated into English. The information was supplemented with notes taken during the discussions. A framework approach was adopted in the coding and analysis of the data to explain the findings of the quantitative survey. Coding was performed manually by two independent investigators. These investigators were an experienced social scientist and a post-graduate student supervised by the social scientist. Although a framework approach was adopted in the analysis, this included content analysis to gauge how dominant perceptions were, to identify emergent themes and to recognize differences in perception between groups in the FGDs. Quotes that typified consensus, or were atypical yet relevant were extracted and are reported here. They are presented here with an indication of the characteristics of the group from which the comment was made (rural or semirural and RDT positive, negative or both).

## Results

### Survey

A total of 3047 caregivers were interviewed in the survey. This fell short of the target 3200 caregivers because some health centres were situated in communities that had less than 100 children under two years of age who lived within 2 kilometres of the centre. The median age of caregivers was 28 years (range 19–45). The majority (76%) were aged between 19 and 35 years. Nearly all (99%) caregivers were mothers who were in either traditional or orthodox marriages. About a third (29%) had had no formal education. Only 50% of caregivers lived in households that had access to piped water. For 676 (22.2%) caregivers the main sources of water were springs, rivers and streams.

Nearly all (98%) caregivers indicated a preference for RDT-based management of malaria over presumptive treatment. The remaining 2% either wanted the diagnosis of malaria to be based on clinical judgement or were unsure of their preference. Compared with caregivers in traditional marriages, caregivers who were single were more likely to consider RDT-based management of malaria acceptable (OR 2.12, 95% CI 1.04–4.33) (Table 1). About 97% of caregivers who preferred RDT-based management of malaria still preferred this approach even after attention had been drawn (in the follow-up question) to the fact that it implied blood draw from their children whenever they visited the health centre with their febrile child. Although 64% of caregivers who preferred RDT-based management indicated they would be worried if they thought their child had malaria and yet were denied ACT (on account of negative RDT results), a greater proportion (76%) of those who rejected the RDT-based case management approach indicated they would be similarly worried (adjusted O.R. 0.57, 95% CI 0.33–0.98). Compared with caregivers who had never secured health insurance, caregivers who had valid (adjusted O.R. 1.30, 95% CI 1.07–1.61) or expired (adjusted O.R. 1.38, 95% CI 1.12–1.73) health insurance were more likely to be worried about their children being denied ACT in the event of an RDT-negative result (Table 2).

**Table 1:** Sociodemographic background of caregivers and the preference for RDT-based management of malaria.

Variable		Preference for RDT-based management of malaria			
Demographic background		Yes	No	O.R. (95% C.I.)	P-value
Age (yrs)	>35	504	11	1.08 (0.37–3.15)	0.94
	19–34	2243	55	0.96 (0.38–2.43	
	<19	212	5	1.00	
Religion	Christian	2355	54	1.40 (0.55–3.55)	0.74
	Moslem	378	10	1.21 (0.41–3.61)	
	Traditionalist	156	5	1.0	
Marital status	Single	655	9	2.12 (1.04–4.33)	0.05
	Orthodox marriage	389	6	1.89 (0.81–4.43)	
	Traditional marriage	1915	56	1.0	
Number of children	One	747	19	0.88 (0.48–1.62)	0.91
	2–3	1184	29	0.91 (0.52–1.59)	
	4 or more	1028	23	1	
Highest educational level	Beyond primary	1417	35	1.14 (0.68–1.94)	0.39
	Primary	692	12	1.63 (0.81–3.28)	
	None	850	24	1.0	
Status in NHIS	Valid insurance	1431	36	0.74 (0.37–1.46)	0.64
	Expired insurance	937	24	0.72 (0.35–1.50)	
	Never insured	591	11	1.0	
Socioeconomic status	Least poor	595	14	1.06 (0.51–2.22)	0.24
	Poor	588	21	0.70 (0.36–1.37)	
	Very poor	600	9	1.67 (0.72–3.84)	
	Most poor	592	12	1.24 (0.57–2.66)	
	Poorest	599	15	1.0	

**Table 2:** Sociodemographic background of caregivers and being worried about ACT denial because of RDT-negative result.

Variable		Worried about ACT denial because of RDT-negative result			
Demographic background		Yes	No	O.R. (95% C.I.)*	P-value
Age (yrs)	>35	182	332	0.98 (0.70–1.36)	0.97
	19–34	807	1489	0.97 (0.72–1.29)	
	<19	78	139	1.0	
Religion	Christian	843	1565	0.73 (0.53–1.01	0.14
	Moslem	132	255	0.70 (0.48–1.02)	
	Traditionalist	68	92	1.0	
Marital status	Single	256	408	1.21 (1.01–1.45)	0.13
	Orthodox marriage	138	257	1.03 (0.82–1.30)	
	Traditional marriage	673	1295	1.0	
Number of children	One	257	509	0.85 (0.70–1.03)	0.20
	2–3	419	794	0.89 (0.75–1.05)	
	4 or more	391	657	1.0	
Highest educational level	Beyond primary	506	945	0.99 (0.83–1.18)	0.81
	Primary	255	448	1.05 (0.86–1.30)	
	None	306	567	1.0	
Status in NHIS	Valid insurance	528	939	1.30 (1.06–1.60)	0.04
	Expired insurance	358	601	1.38 (1.11–1.72)	
	Never insured	181	420	1.0	
Socioeconomic status	Least poor	195	413	0.98 (0.77–1.24)	0.09
	Poor	221	387	1.18 (0.93–1.50)	
	Very poor	228	381	1.24 (0.98–1.57)	
	Most poor	228	375	1.26 (0.99–1.59)	
	Poorest	200	414	1.0	
Accept RDT-based management of malaria	Yes	1916	1055	0.57 (0.33–0.99)	0.05
	No	54	17		

* Unadjusted odds ratios.

### Focus group discussions

Forty-six caregivers participated in the six FGDs that were conducted. Each FGD was made up of 6–8 caregivers who lived around one health centre. Health centres were purposively sampled to obtain a mix of centres sited in typically rural and semi-rural settings. All discussants were the biological mothers of the children they had sent to the health centre in the preceding 6 weeks. The median age of caregivers was 28 years (range 19–45).

### Factors positively influencing acceptability

Based upon the FGDs, the factors that positively influenced caregivers to prefer RDT-based management of malaria were: the perception that it represented improvement in the quality of care at the health centre level; the belief that it provided objective assessment of what was wrong with the child; led to favourable clinical outcomes; and afforded opportunity for interaction between the health workers and caregivers.

Most caregivers were highly enthusiastic about the availability of a test for malaria at the health centres. They frequently referred to the RDT kit as a “machine” that was not available previously but is now available. Its availability was perceived to represent improvement in the quality of services delivered at the health centres in terms of both accurate diagnosis and treatment.

*“What I am very happy about it that at first it was in the big hospital that they would take the child's blood to see what is in it. Even there at times they won't take it but now they do it here and it's good for them to do it to know what is wrong with the child. That is what I am happy about!”.*

*[RDT-Negatives, rural].*

*“At first because there was no blood testing they weren't giving proper medicines to the children”.*

*[RDT-Negatives, semirural].*

Most caregivers agreed with the notion that the blood test result provided objective evidence on the condition of the child. It was felt that this relieved them (caregivers) of the burden of responsibility when reporting the symptoms of the child at the facility.

*“I want him [fieldworker] to take the blood and use the machine so tell what the child's problem is. If I were to say what the problem was, I could get it wrong”.*

*[RDT- positives & Negatives, semirural].*

*“After the test he [fieldworker] writes the result on the paper and sends it to the doctor. That way, when you meet the doctor, you don't have too much to say. What is written on the paper says it all for you”.*

*[RDT-Positives & Negatives, semirural].*

Favourable clinical outcome was perceived by many caregivers to be an outcome of use of the “machine” to guide prescriptions.

*“Previously whenever I brought the child to the health centre, they will give her medicine without any test and the child remained ill. Now they take her blood and do the test before saying what the child's problem is. With that when they give medicines, the child gets well”.*

[RDT-Positives, *semirural*].

The lucidity and enthusiasm with which caregivers described the procedures of the test suggested a high level of interest. The enthusiasm extended to the blood test that was used to determine haemoglobin levels. It also pointed to an appreciation of the level of interaction between themselves (caregivers) and the fieldworkers who conducted the test.

*“Whenever two lines appear, it means there is malaria. When only one line appears, it means there is no malaria”.*

[RDT Positives and negatives, semirural].

*“He (fieldworker) uses it to check if you have malaria. It is like the one they use in pregnant women. If he drops the blood on the paper and two lines appear it means you have malaria, but if only one line appears, it means you don't have malaria. I asked about it and he showed it to me”.*

[RDT-Negatives, rural].

The awareness among some caregivers that fever could be due to causes other than malaria, and how RDT made such distinction clearer, also promoted a positive appreciation of RDT-based management of malaria.

*“At first we thought that all fever was caused by malaria but now that they use the test we know otherwise”.*

[RDT-Positives, rural].

*“I like the test because the fever might not be due to malaria. It could due to teething or something else. You may think it is malaria but the test may show otherwise”.*

[RDT-positives, rural].

*“When he takes the blood, he puts it in a glass and puts a paper on it and puts it in a machine before he tells you if the blood has reduced or not”.*

[RDT-positive and negatives, rural].

### Factors negatively influencing acceptability

The few caregivers who had misgivings about RDT-based management of malaria raised issues related to the effect of blood draw and apprehension that the test could rather be a test for HIV. Comments to these effects were however few.

*“When I went there and he said he was checking the blood I wasn't happy because the child was sick. When I looked at beneath the child's eyes, I saw that it was white (pale) and I didn't know how he (fieldworker) was not going to be able to replace the blood that he was taking”.*

[RDT-negatives, rural].

*“On my visit, I was worried about the quantity of blood that was being drawn, and how it will be replaced in the body. However the fieldworker explained it to me and I accepted it”.*

[RDT-negative, rural].

*“I haven't personally asked the worker to explain it to me. But I learnt that part of the lines shows red and part shows green. If it shows red, it is AIDS but if it shows green then it is malaria. That is my mind”.*

[RDT-positives, semirural].

Rejection of RDT-based management of malaria was also influenced by caregivers' pre-conceived notions of what could be the cause of a child's illness. The dominant notion was that the presence of fever was necessarily indicative of malaria and therefore warranted malaria treatment. In addition caregivers' prior knowledge that a child had been bitten by a mosquito in the days leading to the illnesses led them to hold to the notion that the child's illness ought to be malaria. A negative RDT result in such instances made caregivers to believe that the result was probably false.

*“I don't trust the result of the RDT because it tends to say there is no malaria even when there high fever”.*

[RDT-positives, semirural].

*“I don't also believe it [RDT] because there are lots of mosquitoes in my room. At times when I wake up from bed, I see blood stains on the bed sheet which is as a results of the mosquito bite but any time I take the child to the hospital they say there is no malaria in the blood. The way the mosquitoes bite us in the room I don't understand why they always say that there is no malaria in the child's blood but what can I do, it is the doctor who has spoken”.*

[Mix of RDT-positive and negatives, rural].

### Acceptance of ACT denial based on an RDT-negative result

Most caregivers did not consider the denial of ACT on account of a negative RDT result a problem. Based on discussions on whether the denial of ACT based on a negative RDT was a problem, the major factors that enhanced acceptance of this strategy were caregiver confidence in the “infallibility” of RDTs, the perception of favourable outcome of treatments that are based on the RDT results, and the knowledge that fever could have other causes.

*“If the machine tells me that is malaria, I believe it is malaria”.*

[RDT-positives, semirural].

*“When my child's RDT test was negative they did not give ACT. They gave other drugs and I thought the child will not recover on those drugs. To my surprise, when I gave the drugs to my child, he recovered”.*

[RDT-negatives, rural].

*“If perhaps I made up my mind that the child had malaria, but when I got here, the machine said the child did not have malaria and therefore they did not give malaria drug, I will accept it because it is the machine that knows better”.*

[RDT-positives, semirural].

Some of the few caregivers who indicated they would be worried if their child was denied ACT said they would adopt alternative ACT-seeking behaviour such as procuring ACT from the drug store.

*“If they don't give me the malaria drugs, I will buy some from the drug store when I get home”.*

[RDT-positives, semirural].

### Attitude of health workers

Across all the six FGDs, caregivers spent a considerable amount of time complaining about the attitude of health workers and how actions such as shouting and blaming caregivers for the ailments of their children made them apprehensive about speaking up during consultation. With each topic in the FGDs caregivers found a way to discuss the negative attitude of health workers. Complaints about the attitude of health workers dominated a significant amount of the discussions overall:

*“At times the way they [health workers] will ask you about the duration of the child's illness is not nice. They appear to be in hurry and won't take their time. She [health worker] will say, you have allowed the child to be sick for a long time. What do you want us to do for the child? [Turning to co-discussants and rhetorically asking] If I knew what could be done for the child, would I have brought the child? Their attitude is very bad”.*

[RDT-positives, semirural].

*“The clinician at the health centre easily gets angry. If he asks you a question and you talk a lot, he gets angry. My child was sick and I explained that the child had fever, cough and rashes. The man got so angry that he didn't write the drugs he was supposed to write for us. He wrote only 2 drugs for me”.*

[RDT-positives & negatives, rural].

*“If you meet a good nurse she would pamper you but if you are not lucky and you meet one who is perhaps already angry from home, she will shout at you. She would be angry as she writes your prescription and accuse you of not taking good care of the child”.*

[RDT-positives, semirural].

Caregivers whose children tested RDT-negative on their last visit and were therefore denied ACT, made comments that reflected a higher level of anxiety about treatment outcome than caregivers whose children tested positive and were therefore given ACT. No clear differences were observed between groups that are resident in rural and semirural areas.

## Discussion

We used a sequential approach to apply both quantitative and qualitative methods to explore the acceptability of RDT-based management of malaria among caregivers in rural Ghana. By using this approach we were able to explain and contextualise some of the findings of the survey, as well as allow for the emergence of new themes during the qualitative study. Although the two methods were applied at different stages of caregivers' experience with RDT-based management of malaria, most of the findings of the qualitative study were consistent with the findings of the survey.

In trying to understand the high level of acceptability of RDT-based management of malaria and its related factors, we find the attitude of caregivers to be consistent with the theory of rational expectation, which states that individuals make choices based on their rational outlook, available information and past experiences [31], [32]. With limited knowledge and exposure to RDT-based management of malaria thus far, caregivers appeared to make the best guess of what this approach means for quality of care.

In terms of rational outlook, caregiver acceptability of RDT-based management of malaria was enhanced by the perception that confirming malaria diagnosis before giving ACT will lead to favourable clinical outcome while management on the basis of clinical judgement alone was speculative and led to unfavourable clinical outcome. Improved clinical outcome is likely to be the major expectation of caregivers as the new strategy is rolled out within the routine health system. Whether their expectations will be met remains to be seen. Currently health care providers cannot, with confidence, assure caregivers that RDT-based management of malaria (while reducing wasteful use of expensive antimalarials) will lead to improved clinical outcome since the scientific evidence for this remains weak [10], [12] and health workers continue to face considerable difficulty in the management of RDT-negative cases [18], [25], [26]. Any perception among caregivers that children who are denied ACT have unfavourable clinical outcome can undermine caregiver confidence in RDT-based malaria treatment. This is also likely to lead to the emergence of alternative ACT-seeking behaviour including the resort to private chemical sellers and other vendors to procure ACTs.

In the context of the clinical trial within which this study was conducted, caregivers appeared quite satisfied with the information fieldworkers provided about RDT-based management of malaria and the procedure adopted. Given that caregivers made very few references to information obtained from other sources, it appears the perception formed of RDT-based management of malaria was based largely on information provided by the fieldworkers and other health workers. As RDT-based management of malaria becomes more firmly established, perceptions of its advantages and disadvantages will be also be influenced by information obtained from other caregivers, family members and other members of the community. Sustaining the apparently high level of acceptability of the intervention will depend how vigorous and consciously health workers educate caregivers about the test and its place in the management of childhood illnesses in general.

In traditional African society, procedures associated with blood draw easily lead to rumours that have the potential to impede adherence to interventions [33], [34]. Suggestions by caregivers that the blood test is for HIV and not malaria have been similarly reported in studies in Uganda and Tanzania [35], [36], [37]. As RDT for malaria is rolled out, the health system will need to be sensitive to community misconceptions about the purpose of the blood draw (e.g. use for HIV test). Allowing the caregivers to watch the procedure and explaining the results as seen on the RDT has the potential to contribute to preventing misunderstandings.

The findings of the study suggest that with appropriate education, test-based management of malaria using RDT can be implemented with very little or no resistance from caregivers in rural Ghana. The high level of acceptance has similarly been reported in studies in Uganda and Tanzania [17], [19] although it has been suggested that the apparently high interest shown by caregivers in the use of RDT in an Ugandan study was more of mere curiosity than a genuine desire that the test outcome be used to influence clinical decision-making [38]. It has to be noted however that the Ugandan study was conducted within the private sector.

The belief that RDT-based management of malaria at primary care level represented improvement in the quality of care was similarly reported in another study in Uganda [35]. This appears to be the major driver of the acceptability of RDT-based management of malaria. It is likely to weigh heavier than counter factors such as the fear of the effect of blood draw and concerns that the test could produce false results. This consideration is consistent with the awareness of caregivers of the fact that blood tests are normally carried out in referral facilities and not health centres. To the extent that referral facilities are generally considered to be superior to health centres, and therefore better equipped to establish causes of illnesses, the extension of blood test to health centres is likely to be perceived to be an improvement in the quality of services.

New themes that emerged from the FGDs provided indication as to other ways through which acceptability of RDT-based management of malaria may be further enhanced. As part of the protocol of the clinical trial, fieldworkers simultaneously conducted rapid blood test to determine haemoglobin level. From the FGDs, it was apparent that this contributed to caregivers' acceptability of RDT-based management of malaria. Another feature that appeared to have left a positive impression on the minds of caregivers was the opportunity the performance of these tests offered for educative interaction between fieldworkers and caregivers. The opportunity for them to be part of the procedure, for example waiting to see the emergence or otherwise of the “two lines” (evidence of positive malaria test) in the window of the test kit was exciting and positive.

### Health Insurance

The finding that caregivers who had health insurance cover (valid or expired) were more likely to be worried if their children were denied ACT suggested an expectation that they were entitled to ACT and the denial of this was the denial of something that was a matter of right. Unfortunately we did not explore this issue further in the focus group discussions. The association between being insured under the national health insurance scheme and accepting the denial of ACT *vis-a-vis* adopting alternative ACT-seeking behaviour should be explored in future research.

### Health worker attitude

The fact that caregivers think the RDT has obviated the need for them to be exact when reporting the symptoms of the child suggest a degree of uneasiness of caregivers when they consult health workers. It was therefore not surprising that caregivers spent a considerable part of the discussions lamenting the negative attitude of health workers. Poor health worker attitude could become the greatest threat to caregivers' acceptability of RDT-based management of malaria and other health interventions in rural communities. Similar issues on health worker attitudes were reported from a study in the neighbouring Ashanti Region of Ghana that was related to the use of RDTs for intermittent screening for malaria in pregnancy [39]. Strategies are urgently needed to improve health worker attitudes if major health interventions are to be well-accepted by the community that intended to benefit from these interventions. Guidelines on the use of RDTs in primary care settings should incorporate the active engagement of caregivers in the procedures of testing and interpreting the results.

### Limitations

The fact that the assessment of caregiver perception of RDT-based management of malaria was conducted within the context of a clinical trial imposes some important limitations on the interpretation of the findings. The studies were conducted at the time when caregivers were either expecting their children to be enrolled into the clinical trial or had their children already enrolled in the trial. Despite assurances to the contrary, it is conceivable that some caregivers had apprehensions about possible negative repercussions for the care of their children if they gave responses or raised issues that were less favourable towards the intervention.

The fieldworkers who performed the RDTs at the health centres received salary from the research centre. They received an uninterrupted supply of all the logistics that were required and also had fewer children to attend to each day. It is reasonable to suppose that these fieldworkers were better motivated than routine health workers. Routine health services in Ghana and other malaria-endemic sub-Saharan African countries are characterized by high patient to health worker ratios, inadequate logistics and lowly-motivated staff [40], [41] who may not interact in the same way with caregivers as the fieldworkers in this study. It is possible therefore that the setting (i.e. clinical trial) contributed to the high level of acceptance experienced in this study.

Although we had constituted groups in the FGDs on the basis of the RDT results of their children during their last visit, and also on the basis of their areas of residence, very little discernible differences were identified during the discussions. This could have been due to the fact that their children had had different RDT results on occasions prior to their last visits.

## Conclusion

Within the context of an on-going clinical trial, we assessed the acceptability of RDT-based management of malaria among caregivers of under-five children in rural Ghana. We found that although RDT-based management of malaria represented a departure from practices that caregivers attending health centres in rural Ghana are used to, the tests were seen as an improvement in the quality of care and therefore acceptable. Acceptability will however be further enhanced if RDT-based management of malaria leads to obvious improvement in clinical outcomes, and tests are conducted with interactive provider education and caregiver participation. Guidelines currently being developed to guide the process of incorporating RDT-based management of malaria into IMCI should consider how caregivers may be actively engaged in the procedures of the test.
